# Novel Interactive Tool for Breast and Ovarian Cancer Risk Assessment (Bright Pink Assess Your Risk): Development and Usability Study

**DOI:** 10.2196/29124

**Published:** 2022-02-24

**Authors:** Elizabeth A Hibler, Angela J Fought, Kiarri N Kershaw, Rebecca Molsberry, Virginia Nowakowski, Deborah Lindner

**Affiliations:** 1 Department of Preventive Medicine Feinberg School of Medicine Northwestern University Chicago, IL United States; 2 Bright Pink Chicago, IL United States

**Keywords:** breast cancer, ovarian cancer, risk assessment, genetic testing

## Abstract

**Background:**

The lifetime risk of breast and ovarian cancer is significantly higher among women with genetic susceptibility or a strong family history. However, current risk assessment tools and clinical practices may identify only 10% of asymptomatic carriers of susceptibility genes. Bright Pink developed the Assess Your Risk (AYR) tool to estimate breast and ovarian cancer risk through a user-friendly, informative web-based quiz for risk assessment at the population level.

**Objective:**

This study aims to present the AYR tool, describe AYR users, and present evidence that AYR works as expected by comparing classification using the AYR tool with gold standard genetic testing guidelines.

**Methods:**

The AYR is a recently developed population-level risk assessment tool that includes 26 questions based on the National Comprehensive Cancer Network (NCCN) guidelines and factors from other commonly used risk assessment tools. We included all women who completed the AYR between November 2018 and January 2019, with the exception of self-reported cancer or no knowledge of family history. We compared AYR classifications with those that were independently created using NCCN criteria using measures of validity and the McNemar test.

**Results:**

There were 143,657 AYR completions, and most participants were either at increased or average risk for breast cancer or ovarian cancer (137,315/143,657, 95.59%). Using our estimates of *increased* and *average* risk as the gold standard, based on the NCCN guidelines, we estimated the sensitivity and specificity for the AYR algorithm–generated risk categories as 100% and 89.9%, respectively (*P*<.001). The specificity improved when we considered the additional questions asked by the AYR to define *increased* risk, which were not examined by the NCCN criteria. By race, ethnicity, and age group; we found that the lowest observed specificity was for the Asian race (85.9%) and the 30 to 39 years age group (87.6%) for the AYR-generated categories compared with the NCCN criteria.

**Conclusions:**

These results demonstrate that Bright Pink’s AYR is an accurate tool for use by the general population to identify women at increased risk of breast and ovarian cancer. We plan to validate the tool longitudinally in future studies, including the impact of race, ethnicity, and age on breast and ovarian cancer risk assessment.

## Introduction

In the United States, there are nearly 250,000 cases of breast cancer and 25,000 cases of ovarian cancer diagnosed annually [1,2]. Breast cancer is the leading cause of cancer-related deaths and the most commonly occurring cancer among women globally [3-5]. In contrast, ovarian cancer is only the 10th most common cancer but the fifth leading cause of cancer-related death among women [1,6] and the leading cause of death from gynecological cancer [7]. Studies have estimated that 5% to 10% of breast cancer and 10% to 18% of ovarian cancer are due to hereditary susceptibility, in particular from breast cancer gene (*BRCA*) mutations or a strong family history [8-10]. Among *BRCA1* carriers, specifically, estimates suggest a 40% to 87% cumulative breast cancer risk by age 70 years, whereas for ovarian cancer risk, estimates range from 16% to 68% [11]. However, studies have also demonstrated that current risk assessment and clinical practices only identify a small proportion of the at-risk population.

A central component of breast and ovarian cancer prevention programs is education on personal risk, as determined by factors such as family history, genetic susceptibility, hormonal risk factors, and modifiable health behaviors [12-14]. However, studies have estimated that current practices identify at most 10% of asymptomatic BRCA 1/2 carriers [8,10,15-17]. Moreover, among those with a strong family history of breast or ovarian cancer, but who have not received genetic testing, an estimated 20% to 30% have a pathogenic mutation in a breast cancer susceptibility gene [10]. Bright Pink is a nonprofit organization devoted to improving breast and ovarian cancer prevention through the belief that empowering educated patients leads to (1) lower distress associated with cancer risk assessment and (2) improved cancer prevention outcomes [18,19].

To achieve these goals, Bright Pink developed the Assess Your Risk (AYR) tool that provides a user-friendly, web-based survey to determine lifetime breast and ovarian cancer risk at the population level. Bright Pink was developed in collaboration with genetic counselors, public health practitioners, and clinicians to ensure alignment with standards of breast and ovarian cancer risk assessment but with a modern, consumer-facing interface that would be accessible to the general population. Since 2014, the Bright Pink AYR has been used by more than 1.2 million women to assess breast and ovarian cancer risk. The AYR tool offers a streamlined, educational, and actionable user experience with the goal of substantially increasing the number of women nationwide, as well as internationally, learning about breast and ovarian cancer risk.

However, the AYR tool has not yet been compared with the gold standard clinical guidelines for identifying women at increased risk for breast and ovarian cancer who are eligible for consultation with a genetic counselor. The aims of this study are to (1) describe the AYR tool and its content, (2) describe the AYR tool user population, and (3) compare the AYR tool classification against the National Comprehensive Cancer Network (NCCN) guidelines for genetic and family high-risk assessment in breast and ovarian cancer [2,17]. Moreover, improving health equity and early identification of young adult women at increased risk are the primary goals of Bright Pink and public health. Thus, this analysis is also the first to examine AYR classification compared with the NCCN criteria by race, ethnicity, and age group. Demonstration that Bright Pink AYR is an accurate tool for risk assessment at the population level is a critical step in ensuring that the general population, as well as patients and providers, have a reliable tool for the identification and education of women at risk for breast and ovarian cancer.

## Methods

### Study Population

Bright Pink, a national nonprofit organization based in Chicago, Illinois, was started in 2007 with the mission of empowering women to know their breast and ovarian cancer risk to manage their health proactively. For this study, eligible participants included Bright Pink AYR 3.0 users from October 2018 to March 2019, who were women aged ≥18 years. We included women of White non-Hispanic or Latinx, African American, Asian, or Hispanic or Latina race and ethnicity and all age groups. We excluded participants with a personal history of breast or ovarian cancer (excluding nonmelanoma skin cancer) or no knowledge of family history from calculations of agreement between the NCCN and the AYR. The goal of the NCCN criteria is to identify individuals who may be eligible for genetic testing and counseling for breast or ovarian cancer; therefore, we included women at increased risk for either breast or ovarian cancer.

### Ethics Approval

This study was approved by the Northwestern University Human Subjects Protection Program (review number STU00207333).

### Bright Pink AYR: History

Bright Pink developed the original AYR tool in 2014 to support the goals of modernizing and increasing access to risk assessment for breast and ovarian cancer at the population level. The AYR tool estimates breast and ovarian cancer risk through a user-friendly, informative web-based quiz with superior-rated readability [20]. In late 2018, Bright Pink introduced a new version of AYR with an updated user experience and the goal of significantly increasing the number of women in the general population learning about breast and ovarian cancer risk. Specifically, the new version of the AYR tool includes a user-friendly quiz experience and updated mobile-first design features based on feedback gathered from surveys and focus groups of key target demographics, including African American women. Bright Pink also updated the educational content throughout the AYR tool to improve the user’s understanding of how their responses affect risk calculations and understand personalized risk management recommendations. The National Society of Genetic Counselors has reviewed and approved the AYR tool. The AYR tool not only provides accessible risk assessment to the general population but also links women to additional resources, including Bright Pink’s Explore Your Genetics website, breast health mobile messaging program, and an online peer support forum.

### Bright Pink AYR: Design and Content

**Figure 1:**
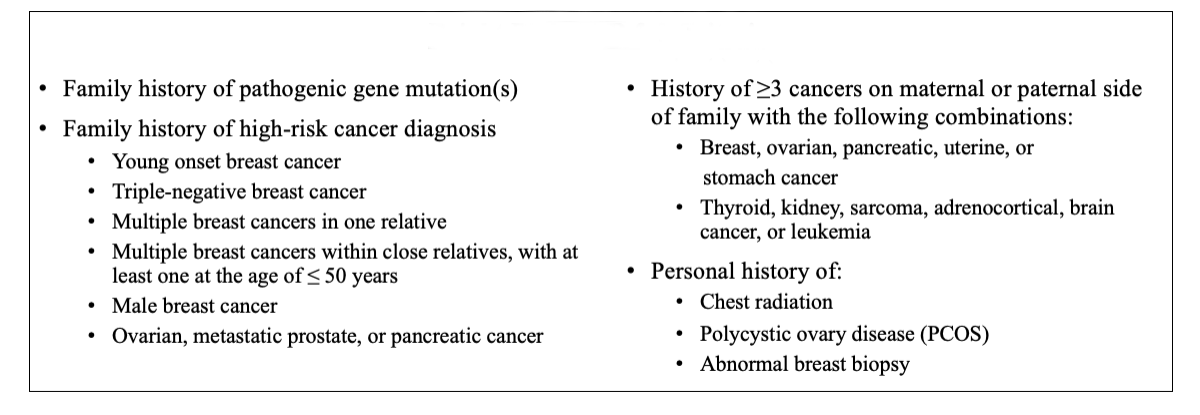
Description of the 3.0 criteria used by the Bright Pink Assess Your Risk (AYR) for breast and ovarian cancer risk assessment among asymptomatic women.

The AYR 3.0 tool includes 26 questions (Figure 1) based on the NCCN criteria for *Genetic/Familial High-Risk Assessment: Breast and Ovarian* [2,17]. However, the AYR tool is unique compared with other existing risk assessment tools designed primarily for use in clinical settings. The NCCN criteria that trigger consideration of genetic testing among asymptomatic individuals include (1) a family history of *BRCA1/2* or other gene variants, (2) a family history of high-risk cancers such as triple-negative or male breast cancer, or (3) a family history of more than 3 cases of cancers on either side.

On the basis of these NCCN criteria, Bright Pink’s AYR tool categorizes women into the *high* category when they report already having a mutation in a breast cancer susceptibility gene identified through genetic testing. Specifically, AYR high risk is triggered by a personal history of positive genetic testing for genes associated with hereditary breast and ovarian cancer syndrome (*BRCA1* and *BRCA2*), Lynch syndrome (*MSH2*, *MLH1*, or *EPCAM*), or Peutz-Jeghers Syndrome (*STK11*) [2]. In contrast, *increased* risk is triggered by a personal history of positive genetic testing for other moderate to lower penetrance mutations (eg, *BRIP1*, *RAD51C*, *RAD51D*, *PMS2*, *and MSH6*) or a relative with a positive gene mutation test combined with no personal history of genetic testing. Moreover, a strong family history of high-risk breast or ovarian cancer, as well as a history of three or more cancers within the family, will trigger an increased risk in the AYR tool. Finally, AYR also uses components from the Gail and Tyrer-Cusick models, including a personal history of childhood radiation to the chest, abnormal breast biopsy, or polycystic ovary syndrome (PCOS) [21-24]. Women with no family history of these genes, and none of the other criteria listed in Figure 1, are categorized as *average* risk and provided with educational messaging focused on appropriate clinical screening guidelines and current evidence related to the impact of health behaviors on cancer prevention.

### Statistical Methods

The primary goal of the AYR is to estimate the lifetime risk of breast and ovarian cancer among women with no history of breast or ovarian cancer. We thus excluded women reporting a previous history of breast or ovarian cancer from the analysis because the focus of AYR is on cancer prevention and early detection. In this subset, we calculated descriptive statistics for the AYR participants overall then by average, increased, and high AYR breast and ovarian categories. We then examined measures of agreement between the NCCN and AYR criteria.

We used the AYR quiz questions to create our own *average* and *increased* categories based on the NCCN criteria for breast and ovarian cancer genetic testing. We then compared these with the categories assigned by the AYR algorithm when the quiz was taken. We assessed measures including sensitivity and specificity to estimate the ability of AYR to distinguish between women who are eligible for genetic testing (AYR increased risk) versus those who are at average risk and thus not eligible for genetic testing, considering our NCCN criteria variables as the *gold standard*. We also examined the positive predictive value (PPV) and negative predictive value (NPV) to determine whether the AYR tool correctly identifies eligible or ineligible women based on NCCN criteria among those in the AYR increased or average categories, respectively. A *true positive* was when AYR risk category matched with the independently created NCCN categories. We calculated McNemar test of agreement, with an *α* of .05.

## Results

### AYR User Population

There were 143,657 AYR completions between October 2018 and March 2019, with complete data on health behaviors and family history. We examined the results for both breast (Table 1) and ovarian (Multimedia Appendix Table S1) cancer AYR risk categories. Overall, most AYR participants reported White race, and the mean age was 30.0 years (SD 10.7), with over half of participants in the 18-29 years age group. The mean BMI was 28.2 (SD 7.6) kg/m^2^, and most women reported that they limit alcohol intake to less than 2 drinks per day and did not smoke. However, 60.54% (86,969/143,657) of women reported that they did not meet the physical activity guidelines for 150 minutes of moderate-intensity activity per week.

**Table 1:** Characteristics of the Bright Pink Assess Your Risk population for breast cancer risk.

Characteristics			Overall (N=143,657)		Average (n=62,760)		Increased (n=74,555)		High (n=6342)
Age (years), mean (SD)			29.66 (10.71)		29.8 (10.76)		30.2 (10.49)		29.0 (12.46)
**Age group (years), n (%)**									
	18-29	84,506 (58.82)		37,689 (60.05)		42,533 (57.05)		4284 (67.55)	
	30-39	32,408 (22.56)		13,558 (21.60)		17,937 (24.06)		913 (14.40)	
	40-49	17,450 (12.15)		7369 (11.74)		9529 (12.78)		552 (8.70)	
	50-64	8152 (5.67)		3647 (5.86)		4050 (5.43)		455 (7.17)	
	≥65	1141 (0.79)		497 (0.79)		506 (0.68)		138 (2.18)	
**Race and ethnicity, n (%)**									
	White	97,227 (67.68)		38,984 (62.12)		53,694 (72.02)		4549 (71.73)	
	African American	5001 (3.48)		2620 (4.17)		2224 (2.98)		157 (2.48)	
	Asian	4469 (3.11)		2924 (4.66)		1398 (1.88)		147 (2.32)	
	Other or multiple	22,411 (15.60)		10,238 (16.31)		11,200 (15.02)		973 (15.34)	
	Ashkenazi Jewish ethnicity	716 (0.50)		249 (0.40)		400 (0.54)		67 (1.06)	
	Hispanic or Latina ethnicity	13,833 (9.62)		7745 (12.34)		5639 (7.56)		449 (7.08)	
BMI, mean (SD)			28.22 (7.63)		28.00 (7.52)		28.51 (7.75)		27.12 (6.99)
Alcohol intake; ≥2 drinks per day, n (%)			16,171 (11.26)		6820 (10.87)		8498 (11.4)0		853 (13.45)
Exercise; <150 min/week, n (%)			86,969 (60.54)		37,481 (59.72)		45,879 (61.45)		3609 (56.91)
Current smoker, n (%)			21,801 (15.18)		8618 (13.73)		12,102 (16.23)		1081 (17.05)
Personal history of breast cancer only, n (%)			4599 (3.20)		0 (0)		0 (0)		4599 (72.52)
Personal history of ovarian cancer only, n (%)			1275 (0.89)		316 (0.50)		948 (1.27)		11 (0.17)
Personal history of breast and ovarian cancer, n (%)			1182 (0.82)		0 (0)		0 (0)		1182 (18.64)
Dense breasts, n (%)			24,347 (16.95)		8448 (13.46)		14,321 (19.21)		1578 (24.88)
History of breastfeeding, n (%)			24,720 (41.50)		10,554 (41.93)		13,304 (41.07)		862 (42.86)
Polycystic ovary syndrome, n (%)			13,825 (9.62)		5116 (8.15)		7881 (10.57)		828 (13.06)
Abnormal biopsy, n (%)			3521 (2.45)		0 (0)		2550 (3.42)		971 (15.31)
Chest radiation, n (%)			1541 (1.07)		0 (0)		1283 (1.72)		258 (4.07)
Family history early-onset breast cancer, n (%)			26,080 (18.15)		0 (0)		23,450 (31.45)		2630 (41.47)
Family history triple-negative breast cancer, n (%)			5851 (4.07)		0 (0)		5267 (7.06)		584 (9.21)
Family history multiple breast cancers in same relative, n (%)			16,453 (11.45)		0 (0)		14,888 (19.97)		1565 (24.68)
Family history of multiple breast cancers with at least one ≤50 years, n (%)			13,721 (9.55)		0 (0)		12,322 (16.53)		1399 (22.06)
Family history of male breast cancer, n (%)			1002 (0.70)		0 (0)		874 (1.17)		128 (2.02)
Family history of ovarian cancer, n (%)			16,711 (11.63)		0 (0)		15,704 (21.06)		1007 (15.88)
Family history of metastatic prostate cancer, n (%)			7813 (5.44)		0 (0)		7481 (10.03)		332 (5.23)
Family history of pancreatic cancer, n (%)			11,732 (8.17)		0 (0)		11,158 (14.97)		574 (9.05)
Personal genetic testing history, n (%)			5290 (3.68)		845 (1.35)		3064 (4.11)		1381 (21.78)
Family member genetic testing history, n (%)			20,553 (24.13)		2103 (6.46)		16,690 (34.26)		1750 (45.44)

We also examined participant characteristics by the risk category assigned by the AYR algorithm, which is shown for breast (Table 1) and ovarian (Multimedia Appendix 1, Table S1) cancers. Most participants were classified as average or increased risk for breast cancer, with only 4.41% (6342/143,657) reporting high risk. White race was the most commonly reported (97,227/143,657, 67.67%) compared with 3.48% (5001/143,657) for African American, 3.11% (4469/143,657) for Asian, and 9.62% (13,833/143,657) for Hispanic or Latina ethnicity. After stratifying for the AYR-assigned risk category, a greater proportion of high-risk women tended to be aged >50 years and reported Ashkenazi Jewish ethnicity. For health behaviors, we observed a trend of higher alcohol intake and current smoking among women with increased and high breast cancer risk AYR categories. However, women in the high-risk category were least likely to report that they did not meet the physical activity guidelines (378/683, 55.3%) compared with 61.89% (49,566/80,091) and 58.53% (36,358/62,122) for increased and average risk, respectively) and reported the lowest mean BMI of 27.1 kg/m^2^. High-risk women were also more likely to have a personal history of dense breast, PCOS, or abnormal breast biopsy as well as a family history of high-risk cancers. For ovarian cancer (Multimedia Appendix 1, Table S1), we observed similar trends overall and by risk category. This is supported by Table S2 in Multimedia Appendix 1, which demonstrates that most participants matched in the AYR risk category for breast and ovarian cancer.

### Agreement Between AYR-Defined Categories Versus Independently Assessed NCCN Risk Categories

In examining the agreement between the AYR tool classifications and our classification based on NCCN criteria for breast and ovarian cancer risk, we calculated the sensitivity, specificity, PPV, and NPV. As shown in Table 2, the sensitivity and NPV were 100% for AYR compared with NCCN for all categories. However, overall, the specificity of the AYR tool was 89.9%, whereas the PPV was 94%. We also examined variation in measures of validity between the AYR and NCCN criteria by race, ethnicity, and age group (Table 3). We found the lowest estimates of specificity and PPV for Asian race, followed by Ashkenazi Jewish ancestry. Overall, specificity ranged from 85.9% for the Asian race to 91.3% for the African American race. For age group, there was also some variation in specificity, and the values ranged from 87.6% for the 30-39 years age group to 93.7% for those aged ≥65 years.

To explore the reasons for the observed variation in specificity and sensitivity, we also examined the frequency of the additional characteristics evaluated by the AYR tool, including personal history of chest radiation, PCOS, or abnormal breast biopsy by race, ethnicity, and age group (Multimedia Appendix 1, Table S3). Women with Ashkenazi Jewish ethnicity reported the highest frequency of previous chest radiation, PCOS, and abnormal breast biopsy. However, Asian women and women reporting for other races also reported 14.88% (638/4288) and 13.93% (2434/17,474) relatively high frequency, respectively, of a history of PCOS. Older women were more likely to report a history of chest radiation, whereas younger women were more likely to report PCOS. We also observed a trend of higher frequency of abnormal breast biopsy for the over 40-49, 50-64, and ≥65 years age groups (905/18,671, 4.85%; 644/7997, 8.05%; and 109/1151, 9.47%, respectively) compared with younger age groups.

**Table 2:** Variation in agreement between National Comprehensive Cancer Network (NCCN) and Assess Your Risk (AYR) by race, ethnicity, and age group.

			AYR compared with NCCN criteria only													
			Total^a^, n (%)		True positive, n		True negative, n		Sensitivity (%)		Specificity, (%)	PPV^b^ (%)		NPV^c^ (%)		*P* value^d^
Overall			117,595 (100)		72,072		40,880		100.00		89.80	93.95		100.00		<.001
**Race and ethnicity**																
	White	82,128 (69.83)		52,078		26,994		100.00		89.83		94.46	100.00		<.001	
	African American	3886 (3.3)		2125		1608		100.00		91.31		93.28	100.00		<.001	
	Asian	3187 (2.71)		1311		1612		100.00		85.93		83.24	100.00		<.001	
	Other	17,614 (14.97)		10,803		6086		100.00		89.36		93.71	100.00		<.001	
	Ashkenazi Jewish	584 (0.49)		373		187		100.00		88.63		93.95	100.00		<.001	
	Hispanic or Latina	10,196 (8.67)		5382		4393		100.00		91.25		92.75	100.00		<.001	
**Age group (years)**																
	18-29	65,352 (55.57)		41,126		21,936		100.00		90.55		94.73	100.00		<.001	
	30-39	28,541 (24.27)		17,475		9694		100.00		87.60		92.72	100.00		<.001	
	40-49	1590 (1.35)		9170		5736		100.00		89.35		93.06	100.00		<.001	
	50-64	7167 (6.09)		3832		3068		100.00		91.99		93.49	100.00		<.001	
	≥65	945 (0.8)		469		446		100.00		93.70		93.99	100.00		<.001	

^a^Sample size excluded women with a history of genetic testing in the high risk category (n=6342), no personal history of cancer (n=7056), or unknown family history (n=19,138). These categories were not mutually exclusive, and 26,062 were excluded from the analysis.

^b^PPV: positive predictive value.

^c^NPV: negative predictive value.

^d^*P* value was calculated using McNemar test.

**Table 3:** Assess Your Risk (AYR) compared with National Comprehensive Cancer Network (NCCN) criteria plus polycystic ovary syndrome, childhood radiation, and abnormal breast biopsy

		AYR full criteria compared with NCCN criteria							
		Total^a^, n (%)	True positive, n	True negative, n	Sensitivity (%)	Specificity (%)	PPV^b^ (%)	NPV^c^ (%)	*P* value^d^
Overall		117,595 (100)	76,714	40,880	100.00	100.00	100.00	100.00	.32
**Race and ethnicity**									
	White	82,128 (69.83)	55,134	26,994	100.00	100.00	100.00	100.00	—^e^
	African American	3886 (3.3)	2277	1608	100.00	99.94	99.96	100.00	.32
	Asian	3187 (2.71)	1575	1612	100.00	100.00	100.00	100.00	—
	Other	17,614 (14.98)	11,528	6086	100.00	100.00	100.00	100.00	—
	Ashkenazi Jewish	584 (0.49)	397	187	100.00	100.00	100.00	100.00	—
	Hispanic or Latina	10,196 (8.67)	5803	4393	100.00	100.00	100.00	100.00	—
**Age group (years)**									
	18-29	65,352 (55.57)	43,416	21,936	100.00	100.00	100.00	100.00	—
	30-39	28,541 (24.27)	18,846	9694	100.00	99.99	99.99	100.00	.32
	40-49	15,590 (13.26)	9854	5736	100.00	100.00	100.00	100.00	—
	50-64	7167 (6.09)	4099	3068	100.00	100.00	100.00	100.00	—
	≥65^d^	945 (8.03)	499	446	100.00	100.00	100.00	100.00	—

^a^Sample size excluded women with a history of genetic testing in the high risk category (n=6342), no personal history of cancer (n=7056), or unknown family history (n=19,138). These categories were not mutually exclusive, and 26,062 were excluded from the analysis.

^b^PPV: positive predictive value.

^c^NPV: negative predictive value.

^d^*P* value was calculated using McNemar test.

^e^No discordant pairs between groups where all measures are 100.00.

## Discussion

### Principal Findings

We present the AYR tool as a valuable, new risk assessment tool with great potential to impact breast and ovarian cancer prevention in the general population. Overall, the AYR tool is highly accurate in identifying women at increased risk for breast and ovarian cancer in comparison with the gold standard NCCN criteria. We also identified differences by race, ethnicity, and age group that highlight opportunities for improving the reach of the AYR tool to additional populations as well as objective for future studies to validate the AYR tool among diverse populations. Overall, the Bright Pink AYR tool provides reliable, evidence-based risk assessment to the general as well as clinical populations for the identification of women at increased risk for breast and ovarian cancer.

### Existing Risk Assessment Tools Compared With Bright Pink AYR

Bright Pink AYR is unique compared with other web-based risk assessment tools for breast and ovarian cancer, primarily because of its nature as a participant or patient-facing web-based tool designed for use by the general population. This includes, but is not limited to, clinical risk assessment tools such as the Breast Cancer Risk Assessment Tool (BCRAT; also known as the Gail model), the International Breast Cancer Intervention Study (IBIS), the Breast and Ovarian Analysis of Disease Incidence and Carrier Estimation Algorithm (BOADICEA) model, and BCRAPRO [25-29]. There is much overlap between these validated, clinically oriented tools with AYR but also important differences. For example, the BCRAT model uses 7 questions including age, race, ethnicity, health history (age at menarche and history of abnormal breast biopsy), and family history of breast cancer to estimate the 5-year risk and lifetime risk of invasive breast cancer based on the probability of cancer incidence during a defined age range [21,22]. The BCRAT model, however, does not include the risk of genetic mutations but instead refers participants to alternative tools for testing [21,22]. In contrast, although software such as IBIS using the Tyrer-Cuzick model also estimates 5-year and lifetime risk, the model incorporates genetic mutations and breast density into the most recent versions [23,24]. Moreover, both IBIS and BOADICEA were recently updated to include polygenetic risk scores, which is a limitation that other models such as the AYR should examine in future iterations. BRCAPRO is a similar model that relies primarily on basic demographics, family history, and genetic testing results, if available [30]. However, one major difference between AYR and these other tools is that, other than BOADICEA, none include lifestyle or health behavior risk factors in the models [27]. Overall, although there are many similarities between AYR and existing breast and ovarian cancer clinical risk assessment tools, the major difference is that AYR was designed as a resource for the general population.

### Opportunities for Improving Risk Assessment Through Bright Pink AYR

Bright Pink built the AYR for the general population as a tool to help inform women of their risk and to provide education on cancer prevention activities appropriate to their risk level. We believe that the patient-oriented interface, developed with the help of marketing experts, makes AYR a particularly user-friendly risk assessment tool with the potential to reach populations that existing clinical tools may not reach because of issues such as health care access. The Bright Pink AYR tool represents an advance in the field of population-level cancer prevention as a screening tool with the potential to improve the reach and use of web-based breast and ovarian risk assessment among diverse populations.

The Bright Pink AYR tool has also demonstrated a strong reach to potentially underserved populations. Epidemiological data from the Surveillance, Epidemiology, and End Results Program demonstrate disparities in breast and ovarian cancer incidence and mortality by race [31-33]. However, studies show that much of the differences in breast and ovarian cancer incidence and mortality rates by race and ethnicity are driven by factors such as access to recommended screening and treatment [34,35]. To improve early cancer detection among high-risk groups and younger women not yet eligible for cancer screening, it is critical to increase access to and use of risk assessment tools in the general population. The Bright Pink AYR was designed to bridge these gaps. However, studies have demonstrated that other current methods for breast and ovarian cancer risk assessment may not currently be acceptable to diverse populations. Studies by Cragun [36] and Sheppard et al [37] demonstrated the disparities by race in the acceptability and use of breast and ovarian cancer risk assessment. The results demonstrate that breast and ovarian cancer risk assessment in a clinical setting is less likely to be acceptable or used by African American and Hispanic or Latina women compared with White women [36,37]. The observed disparities by race and ethnicity in breast and ovarian cancer risk as well as use of risk assessment tools justify the need to modernize risk assessment to make it more broadly accessible to the general population. We have demonstrated that the Bright Pink AYR reaches these diverse populations in the general population and provides accurate risk assessment; however, the educational follow-up emails also make AYR unique.

The AYR tool is also unique compared with other tools in that health behaviors are collected and used to tailor email messages to promote cancer prevention behaviors. Previous studies have demonstrated the impact of education on health behaviors among high-risk populations. For example, Quach et al [38] examined baseline and 6-month reporting of health behaviors among Ashkenazi Jewish individuals (n=120) who underwent genetic testing for *BRCA1/2*. The study found that women and those with higher education were more likely to report a healthy diet. The results showed no change in diet, vitamin use, or physical activity over time. However, there was no education component for health behaviors incorporated into the genetic counseling sessions. Another study examined health behaviors among participants of self-reported genetic testing (n=3016; with n=136 reporting genetic testing) using data from the Health Information National Trends Survey 4 [39]. The results demonstrated that lifestyle factors were not statistically significantly different between those who reported genetic testing and those who did not. However, the sample size was relatively small, and the study did not examine multivariate models. The AYR tool collects data on health behaviors, unlike many other clinician-oriented risk assessment tools. However, the AYR educational component has a strong potential to impact population-level participation in cancer prevention activities.

Studies have examined the impact of risk assessment programs for breast or ovarian cancer on adherence to cancer prevention guidelines or recommendations for lifestyle risk factors, and most have identified opportunities for improvement [38-43]. For women with a strong family history, Price et al [44] reported that among 748 women in a breast cancer family registry, between baseline and 3-year surveys, 16% were underscreened for mammography and 55% were underscreened for clinical breast examinations. Moreover, a study by Loescher et al [45] examined cancer surveillance behaviors among 107 women aged ≥18 years who presented for genetic risk assessment for breast and ovarian cancer. The results showed that 60% engaged in the minimal level of recommended breast cancer prevention activities, but 70% reported behaviors below optimal guidelines for cancer prevention. The study also found that a lack of physician recommendation was the most commonly reported reason for not engaging in breast or cancer prevention activities [45]. Similarly, Botkin et al [46] studied the impact of *BRCA1* genetic testing on preventative cancer screening behavior over 2 years (n=408). Among women aged ≥40 years, 82% of mutation carriers followed guidelines for screening mammography in the first year and 67% in the second year, which was significantly increased from baseline and greater than the levels observed among noncarriers [46]. These studies provide evidence that knowledge of risk for breast and ovarian cancer may not only impact participation in cancer prevention activities but also that reported levels are not optimal and expanded adoption of combined risk assessment and educational modalities such as the AYR tool may improve cancer prevention in the general population.

There are strengths and limitations of the current analysis. The strengths include the very large sample size of the completions of the AYR tool in a short period and a diverse population. However, although the population was diverse, the frequency by race and ethnicity was lower than expected for some populations. This reflects the opportunity to improve access and use of the AYR tool in diverse populations. Moreover, the questions asked by the Bright Pink AYR tool are categorical and often binomial, such as health or family history and physical activity questions. This limits the level of detailed data collected on high-risk cancers and the ability to evaluate factors such as age at cancer diagnosis and pathology or subtype of breast cancer. Finally, these data were cross-sectional. Longitudinal studies will be necessary to validate the accuracy of AYR risk assessment in breast and ovarian cancer risk prevention. However, overall, the results demonstrate that AYR accurately classifies women according to breast and ovarian cancer risk using existing gold standard criteria.

These results demonstrate that Bright Pink’s AYR accurately classifies women at an increased risk of breast or ovarian cancer. The variation observed by race, ethnicity, and age group demonstrates the need to improve access and use of risk assessment tools in diverse populations. Overall, Bright Pink AYR is a valuable tool for use by the general population as well as patients and clinical providers for early detection and education, which will improve the prevention of breast and ovarian cancer.

## Appendix

Multimedia Appendix 1 Supplemental tables describing the Bright Pink Assess Your Risk population and characteristics.

## Abbreviations

AYR: Assess Your Risk
BCRAT: Breast Cancer Risk Assessment Tool
BOADICEA: Breast and Ovarian Analysis of Disease Incidence and Carrier Estimation Algorithm
IBIS: International Breast Cancer Intervention Study
NCCN: National Comprehensive Cancer Network
NPV: negative predictive value
PCOS: polycystic ovary syndrome
PPV: positive predictive value

## References

[1] Siegel RL, Miller KD, Jemal A (2016). Cancer statistics, 2016. CA Cancer J Clin.

[2] Daly MB, Pilarski R, Berry M, Buys SS, Farmer M, Friedman S, Garber JE, Kauff ND, Khan S, Klein C, Kohlmann W, Kurian A, Litton JK, Madlensky L, Merajver SD, Offit K, Pal T, Reiser G, Shannon KM, Swisher E, Vinayak S, Voian NC, Weitzel JN, Wick MJ, Wiesner GL, Dwyer M, Darlow S (2017). NCCN guidelines insights: genetic/familial high-risk assessment: breast and ovarian, version 2.2017. J Natl Compr Canc Netw.

[3] DeSantis CE, Bray F, Ferlay J, Lortet-Tieulent J, Anderson BO, Jemal A (2015). International variation in female breast cancer incidence and mortality rates. Cancer Epidemiol Biomarkers Prev.

[4] Ferlay J, Soerjomataram I, Dikshit R, Eser S, Mathers C, Rebelo M, Parkin DM, Forman D, Bray F (2015). Cancer incidence and mortality worldwide: sources, methods and major patterns in GLOBOCAN 2012. Int J Cancer.

[5] Tao Z, Shi A, Lu C, Song T, Zhang Z, Zhao J (2015). Breast cancer: epidemiology and etiology. Cell Biochem Biophys.

[6] Fitzmaurice C (2018). Global, regional, and national cancer incidence, mortality, years of life lost, years lived with disability, and disability-adjusted life-years for 29 cancer groups, 2006 to 2016: a systematic analysis for the Global Burden of Disease study. J Clin Oncol.

[7] Grossman DC, Curry SJ, Owens DK, Barry MJ, Davidson KW, Doubeni CA, Epling JW, Kemper AR, Krist AH, Kurth AE, Landefeld CS, Mangione CM, Phipps MG, Silverstein M, Simon MA, Tseng CW, US Preventive Services Task Force (2018). Screening for ovarian cancer: US preventive services task force recommendation statement. J Am Med Assoc.

[8] Guo F, Hirth JM, Lin Y, Richardson G, Levine L, Berenson AB, Kuo Y (2017). Use of BRCA Mutation Test in the U.S., 2004-2014. Am J Prev Med.

[9] Oosterwijk JC, de Vries J, Mourits MJ, de Bock GH (2014). Genetic testing and familial implications in breast-ovarian cancer families. Maturitas.

[10] Afghahi A, Kurian AW (2017). The changing landscape of genetic testing for inherited breast cancer predisposition. Curr Treat Options Oncol.

[11] Kuchenbaecker KB, Hopper JL, Barnes DR, Phillips K, Mooij TM, Roos-Blom M, Jervis S, van Leeuwen FE, Milne RL, Andrieu N, Goldgar DE, Terry MB, Rookus MA, Easton DF, Antoniou AC, McGuffog L, Evans DG, Barrowdale D, Frost D, Adlard J, Ong K, Izatt L, Tischkowitz M, Eeles R, Davidson R, Hodgson S, Ellis S, Nogues C, Lasset C, Stoppa-Lyonnet D, Fricker J, Faivre L, Berthet P, Hooning MJ, van der Kolk LE, Kets CM, Adank MA, John EM, Chung WK, Andrulis IL, Southey M, Daly MB, Buys SS, Osorio A, Engel C, Kast K, Schmutzler RK, Caldes T, Jakubowska A, Simard J, Friedlander ML, McLachlan S, Machackova E, Foretova L, Tan YY, Singer CF, Olah E, Gerdes A, Arver B, Olsson H, BRCA1 and BRCA2 Cohort Consortium (2017). Risks of breast, ovarian, and contralateral breast cancer for BRCA1 and BRCA2 mutation carriers. J Am Med Assoc.

[12] Chen S, Iversen ES, Friebel T, Finkelstein D, Weber BL, Eisen A, Peterson LE, Schildkraut JM, Isaacs C, Peshkin BN, Corio C, Leondaridis L, Tomlinson G, Dutson D, Kerber R, Amos CI, Strong LC, Berry DA, Euhus DM, Parmigiani G (2006). Characterization of BRCA1 and BRCA2 mutations in a large United States sample. J Clin Oncol.

[13] Hoskins KF, Zwaagstra A, Ranz M (2006). Validation of a tool for identifying women at high risk for hereditary breast cancer in population-based screening. Cancer.

[14] Rockhill B, Spiegelman D, Byrne C, Hunter DJ, Colditz GA (2001). Validation of the Gail et al. model of breast cancer risk prediction and implications for chemoprevention. J Natl Cancer Inst.

[15] Shiovitz S, Korde LA (2015). Genetics of breast cancer: a topic in evolution. Ann Oncol.

[16] Crawford B, Adams SB, Sittler T, van den Akker J, Chan S, Leitner O, Ryan L, Gil E, van 't Veer L (2017). Multi-gene panel testing for hereditary cancer predisposition in unsolved high-risk breast and ovarian cancer patients. Breast Cancer Res Treat.

[17] Daly MB, Pilarski R, Axilbund JE, Berry M, Buys SS, Crawford B, Farmer M, Friedman S, Garber JE, Khan S, Klein C, Kohlmann W, Kurian A, Litton JK, Madlensky L, Marcom PK, Merajver SD, Offit K, Pal T, Rana H, Reiser G, Robson ME, Shannon KM, Swisher E, Voian NC, Weitzel JN, Whelan A, Wick MJ, Wiesner GL, Dwyer M, Kumar R, Darlow S (2016). Genetic/familial high-risk assessment: breast and ovarian, version 2.2015. J Natl Compr Canc Netw.

[18] Bjørnslett M, Dahl AA, Sørebø O, Dørum A (2015). Psychological distress related to BRCA testing in ovarian cancer patients. Fam Cancer.

[19] Graves KD, Vegella P, Poggi EA, Peshkin BN, Tong A, Isaacs C, Finch C, Kelly S, Taylor KL, Luta G, Schwartz MD (2012). Long-term psychosocial outcomes of BRCA1/BRCA2 testing: differences across affected status and risk-reducing surgery choice. Cancer Epidemiol Biomarkers Prev.

[20] Cortez S, Milbrandt M, Kaphingst K, James A, Colditz G (2015). The readability of online breast cancer risk assessment tools. Breast Cancer Res Treat.

[21] Costantino JP, Gail MH, Pee D, Anderson S, Redmond CK, Benichou J, Wieand HS (1999). Validation studies for models projecting the risk of invasive and total breast cancer incidence. J Natl Cancer Inst.

[22] Gail MH, Brinton LA, Byar DP, Corle DK, Green SB, Schairer C, Mulvihill JJ (1989). Projecting individualized probabilities of developing breast cancer for white females who are being examined annually. J Natl Cancer Inst.

[23] Brentnall AR, Harkness EF, Astley SM, Donnelly LS, Stavrinos P, Sampson S, Fox L, Sergeant JC, Harvie MN, Wilson M, Beetles U, Gadde S, Lim Y, Jain A, Bundred S, Barr N, Reece V, Howell A, Cuzick J, Evans DGR (2015). Mammographic density adds accuracy to both the Tyrer-Cuzick and Gail breast cancer risk models in a prospective UK screening cohort. Breast Cancer Res.

[24] Brentnall AR, Cuzick J, Buist DS, Bowles EJ (2018). Long-term accuracy of breast cancer risk assessment combining classic risk factors and breast density. JAMA Oncol.

[25] Daly MB, Axilbund JE, Buys S, Crawford B, Farrell CD, Friedman S, Garber JE, Goorha S, Gruber SB, Hampel H, Kaklamani V, Kohlmann W, Kurian A, Litton J, Marcom PK, Nussbaum R, Offit K, Pal T, Pasche B, Pilarski R, Reiser G, Shannon KM, Smith JR, Swisher E, Weitzel JN, National Comprehensive Cancer Network (2010). Genetic/familial high-risk assessment: breast and ovarian. J Natl Compr Canc Netw.

[26] Nelson H, Huffman L, Fu R, Harris E, Walker M, Bougatsos C (2005). Genetic risk assessment and BRCA mutation testing for breast and ovarian cancer susceptibility. U.S. Preventive Services Task Force Evidence Syntheses, formerly Systematic Evidence Reviews.

[27] Terry M, Liao Y, Whittemore A, Leoce N, Buchsbaum R, Zeinomar N (2019). 10-year performance of four models of breast cancer risk: a validation study. Lancet Oncol.

[28] Choudhury PP, Brook MN, Hurson AN, Lee A, Mulder CV, Coulson P, Schoemaker MJ, Jones ME, Swerdlow AJ, Chatterjee N, Antoniou AC, Garcia-Closas M (2021). Comparative validation of the BOADICEA and Tyrer-Cuzick breast cancer risk models incorporating classical risk factors and polygenic risk in a population-based prospective cohort of women of European ancestry. Breast Cancer Res.

[29] Choudhury PP, Wilcox AN, Brook MN, Zhang Y, Ahearn T, Orr N, Coulson P, Schoemaker MJ, Jones ME, Gail MH, Swerdlow AJ, Chatterjee N, Garcia-Closas M (2020). Comparative validation of breast cancer risk prediction models and projections for future risk stratification. J Natl Cancer Inst.

[30] Berry DA, Iversen ES, Gudbjartsson DF, Hiller EH, Garber JE, Peshkin BN, Lerman C, Watson P, Lynch HT, Hilsenbeck SG, Rubinstein WS, Hughes KS, Parmigiani G (2002). BRCAPRO validation, sensitivity of genetic testing of BRCA1/BRCA2, and prevalence of other breast cancer susceptibility genes. J Clin Oncol.

[31] Hung M, Ekwueme DU, Rim SH, White A (2016). Racial/ethnicity disparities in invasive breast cancer among younger and older women: an analysis using multiple measures of population health. Cancer Epidemiol.

[32] DeSantis CE, Fedewa SA, Goding SA, Kramer JL, Smith RA, Jemal A (2015). Breast cancer statistics, 2015: convergence of incidence rates between black and white women. CA Cancer J Clin.

[33] Edwards BK, Noone A, Mariotto AB, Simard EP, Boscoe FP, Henley SJ, Jemal A, Cho H, Anderson RN, Kohler BA, Eheman CR, Ward EM (2014). Annual Report to the Nation on the status of cancer, 1975-2010, featuring prevalence of comorbidity and impact on survival among persons with lung, colorectal, breast, or prostate cancer. Cancer.

[34] Bandera EV, Lee VS, Rodriguez-Rodriguez L, Powell CB, Kushi LH (2016). Racial/ethnic disparities in ovarian cancer treatment and survival. Clin Cancer Res.

[35] Freeman HP (2006). Patient navigation: a community based strategy to reduce cancer disparities. J Urban Health.

[36] Cragun D, Weidner A, Lewis C, Bonner D, Kim J, Vadaparampil ST, Pal T (2017). Racial disparities in BRCA testing and cancer risk management across a population-based sample of young breast cancer survivors. Cancer.

[37] Sheppard VB, Mays D, LaVeist T, Tercyak KP (2013). Medical mistrust influences black women's level of engagement in BRCA 1/2 genetic counseling and testing. J Natl Med Assoc.

[38] Quach J, Porter K, Leventhal H, Kelly KM (2009). Health behaviors among Ashkenazi Jewish individuals receiving counseling for BRCA1 and BRCA2 mutations. Fam Cancer.

[39] Quillin JM (2016). Lifestyle risk factors among people who have had cancer genetic testing. J Genet Couns.

[40] Forman A, Hall M (2009). Influence of race/ethnicity on genetic counseling and testing for hereditary breast and ovarian cancer. Breast J.

[41] Simon MS, Petrucelli N (2009). Hereditary breast and ovarian cancer syndrome : the impact of race on uptake of genetic counseling and testing. Methods Mol Biol.

[42] Shulman LP (2010). Hereditary breast and ovarian cancer (HBOC): clinical features and counseling for BRCA1 and BRCA2, Lynch syndrome, Cowden syndrome, and Li-Fraumeni syndrome. Obstet Gynecol Clin North Am.

[43] Kurian AW (2010). BRCA1 and BRCA2 mutations across race and ethnicity: distribution and clinical implications. Curr Opin Obstet Gynecol.

[44] Price MA, Butow PN, Charles M, Bullen T, Meiser B, McKinley JM, McLachlan S, Phillips K, kConFab PsychosocialClinical Follow-Up Groups, kConFab Investigators (2010). Predictors of breast cancer screening behavior in women with a strong family history of the disease. Breast Cancer Res Treat.

[45] Loescher LJ, Lim KH, Leitner O, Ray J, D'Souza J, Armstrong CM (2009). Cancer surveillance behaviors in women presenting for clinical BRCA genetic susceptibility testing. Oncol Nurs Forum.

[46] Botkin JR, Smith KR, Croyle RT, Baty BJ, Wylie JE, Dutson D, Chan A, Hamann HA, Lerman C, McDonald J, Venne V, Ward JH, Lyon E (2003). Genetic testing for a BRCA1 mutation: prophylactic surgery and screening behavior in women 2 years post testing. Am J Med Genet A.

