# Supplementary material for: Novel Interactive Tool for Breast and Ovarian Cancer Risk Assessment (Bright Pink Assess Your Risk): Development and Usability Study
Source: J Med Internet Res. 2022 Feb 24;24(2):e29124. doi: 10.2196/29124 (PMC8914739; doi:10.2196/29124)
Supplement: Multimedia Appendix 1 [file jmir_v24i2e29124_app1.docx]

**Table S1.** Characteristics of the Bright Pink Assess Your Risk population for ovarian cancer risk.

| Characteristic | Overall  (N=142, 896) | Average  (N=62,122) | Increased  (N=80, 091) | High  (N=683) |
| --- | --- | --- | --- | --- |
| **Age (years), mean (SD)** | 29.98 (10.71) | 29.72 (11.03) | 30.00 (10.39) | 32.17 (10.66) |
| **Age group (years), n (%)** |  |  |  |  |
| 18-29 | 84,494 (59.13) | 37,875 (60.97) | 46,302 (57.81) | 317 (46.41) |
| 30-39 | 32,126 (22.48) | 12,705 (20.45) | 19,194 (23.97) | 227 (33.24) |
| 40-49 | 17,151 (12.00) | 7133 (11.48) | 9932 (12.40) | 86 (12.59) |
| 50-64 | 7990 (5.59) | 3825 (6.16) | 4116 (5.14) | 49 (7.17) |
| ≥65 | 1135 (0.79) | 584 (0.94) | 547 (0.68) | 4 (0.59) |
| **Race-ethnicity, n (%)** |  |  |  |  |
| White | 96,580 (67.59) | 38,786 (62.44) | 57,335 (71.59) | 459 (67.20) |
| African American | 10,496 (7.35) | 5474 (8.81) | 4997 (6.24) | 24 (3.51) |
| Asian | 4473 (3.13) | 2771 (4.46) | 1691 (2.11) | 11 (1.61) |
| Other or multiple | 16,839 (11.78) | 7190 (12.30) | 9524 (11.89) | 125 (18.30) |
| Ashkenazi Jewish ethnicity | 713 (0.50) | 260 (0.42) | 419 (0.52) | 34 (4.98) |
| Hispanic or Latinx ethnicity | 13,795 (9.65) | 7640 (12.30) | 6125 (7.65) | 30 (4.39) |
| BMI, mean (SD) | 28.20 (7.62) | 27.54 (7.25) | 28.73 (7.89) | 27.29 (7.00) |
| Alcohol intake, 2+ drinks per day, n (%) | 16,018 (11.21) | 6962 (11.21) | 8949 (11.17) | 107 (15.67) |
| Exercise, <150 minutes per week, n (%) | 86,302 (60.39) | 36,358 (58.53) | 49,566 (61.89) | 378 (55.34) |
| Current smoker, n (%) | 21,849 (15.29) | 8672 (13.96) | 13,064 (16.31) | 113 (16.54) |
| Personal history of breast cancer, n (%) | 4579 (3.20) | 1065 (1.71) | 3438 (4.29) | 76 (11.13) |
| Personal history of ovarian cancer, n (%) | 1276 (0.89) | 312 (0.50) | 952 (1.19) | 12 (1.76) |
| History of breast and ovarian cancer, n (%) | 1182 (0.83) | 90 (0.14) | 1056 (1.32) | 36 (5.27) |
| Dense breasts, n (%) | 23,965 (16.77) | 8553 (13.77) | 15,089 (18.84) | 323 (47.29) |
| History of breastfeeding, n (%) | 23,959 (16.77) | 9933 (15.99) | 13,864 (17.31) | 162 (23.72) |
| History of polycystic ovary syndrome, n (%) | 13,825 (9.67) | 0 (0.0) | 13,725 (17.14) | 100 (14.64) |
| History of abnormal biopsy, n (%) | 3492 (2.44) | 1017 (1.64) | 2414 (3.01) | 61 (8.93) |
| History of chest radiation, n (%) | 1530 (1.07) | 620 (1.00) | 872 (1.09) | 38 (5.56) |
| Family history of young onset breast cancer, n (%) | 26,080 (18.09) | 0 (0.0) | 25,699 (32.09) | 381 (55.78) |
| Family history of triple-negative breast cancer, n (%) | 5851 (4.06) | 0 (0.0) | 5748 (7.18) | 103 (15.08) |
| Family history of multiple breast cancers in same relative, n (%) | 16,453 (11.41) | 0 (0.0) | 16,229 (20.26) | 224 (32.80) |
| Family history of multiple  breast cancers in family, at  least one ≤50 years, n (%) | 13,721 (9.52) | 0 (0.0) | 13,479 (16.83) | 242 (35.43) |
| Family history of male breast cancer, n (%) | 1002 (0.70) | 0 (0.0) | 961 (1.20) | 41 (6.00) |
| Family history of ovarian cancer, n (%) | 16,711 (11.59) | 0 (0.0) | 16,499 (20.60) | 212 (31.04) |
| Family history of metastatic prostate cancer, n (%) | 7813 (5.42) | 0 (0.0) | 7765 (9.70) | 48 (7.03) |
| Family history of pancreatic cancer, n (%) | 11,732 (8.14) | 0 (0.0) | 11,649 (14.54) | 83 (12.15) |
| Genetic testing (self), n (%) | 5272 (3.69) | 1041 (1.68) | 3548 (4.43) | 683 (100.0) |
| Genetic testing (family member), n (%) | 20,512 (16.61) | 2204 (4.98) | 17,805 (22.68) | 503 (76.44) |

**Table S2.** Overlap between breast and ovarian cancer AYR risk assessment categories.

| Ovarian cancer category | Breast cancer category, n (%) | | | |
| --- | --- | --- | --- | --- |
|  | Average | Increased | High | Total |
|  |  |  |  |  |
| Average | 56,663 (39.74) | 3976 (2.79) | 1155 (0.81) | 61,794 (43.34) |
| Increased | 5120 (3.59) | 70,451 (49.42) | 4520 (3.17) | 80,091 (56.18) |
| High | 3 (<0.1) | 33 (0.02) | 647 (0.45) | 683 (0.48) |
| Total | 61,786 (43.34) | 74,460 (52.23) | 6322 (4.43) | 142,568 |

**Table S3**. Frequency of additional factors considered in the AYR risk assessment tool by race-ethnicity and age group.

|  | Total | Additional factors in AYR, n (%) | | | |  | |
| --- | --- | --- | --- | --- | --- | --- | --- |
|  |  | Chest radiation | Polycystic ovary syndrome | Abnormal breast biopsy | Total | |  |
| **Race-ethnicity** |  |  |  |  |  | |  |
| White | 102,492 | 1,268 (1.24) | 12,168 (11.87) | 3,165 (3.09) | 16,601 (16.20) | |  |
| African American | 10,402 | 165 (1.59) | 880 (8.46) | 254 (2.44) | 1,299 (12.49) | |  |
| Asian | 4,288 | 77 (1.80) | 638 (14.88) | 96 (2.24) | 811 (18.91) | |  |
| Other or multiple | 17,474 | 291 (1.67) | 2,434 (13.93 | 505 (2.89) | 3,230 (18.48) | |  |
| Ashkenazi Jewish  ethnicity | 750 | 24 (2.6) | 105 (11.5) | 49 (5.4) | 178 (23.7) | |  |
| Hispanic or Latina  ethnicity | 13,415 | 244 (1.82) | 1,462 (10.90) | 337 (2.51) | 2,043 (15.23) | |  |
| **Age group (years)** |  |  |  |  |  | |  |
| 18-29 | 85,455 | 1,238 (1.45) | 10,030 (11.74) | 1,986 (2.32) | 13,254 (15.51) | |  |
| 30-39 | 35,547 | 463 (1.30) | 5,161 (14.52) | 752 (2.12) | 6,376 (17.94) | |  |
| 40-49 | 18,671 | 212 (1.14) | 2,060 (11.03) | 905 (4.85) | 3,177 (17.02) | |  |
| 50-64 | 7,997 | 127 (1.59) | 418 (5.23) | 644 (8.05) | 1,189 (14.87) | |  |
| ≥65 | 1,151 | 30 (2.61) | 18 (1.56) | 109 (9.47) | 157 (13.64) | |  |
